# Pentraxin 3 is more accurate than C-reactive protein for Takayasu arteritis activity assessment: A systematic review and meta-analysis

**DOI:** 10.1371/journal.pone.0245612

**Published:** 2021-02-02

**Authors:** Xiaoting Wen, Ruihong Hou, Ke Xu, Yunxia Han, Junping Hu, Yan Zhang, Yazhen Su, Jinfang Gao, Gailian Zhang, Liyun Zhang

**Affiliations:** 1 Department of Rheumatology, Shanxi Bethune Hospital, Shanxi Academy of Medical Sciences, Taiyuan, Shanxi, China; 2 Department of Rheumatology, Shanxi Bethune Hospital Affiliated to Shanxi Medical University, Taiyuan, Shanxi, China; 3 Department of Clinical Laboratory, Shanxi Bethune Hospital, Shanxi Academy of Medical Sciences, Taiyuan, Shanxi, China; Nippon Medical School Institute for Advanced Medical Sciences, JAPAN

## Abstract

**Aims:**

Whether the circulating levels of pentraxin 3 (PTX3), an acute phase reactant (APR), are higher in active Takayasu arteritis (TAK), and if so, whether PTX3 is more accurate than C-reactive protein (CRP) in TAK activity assessment has been investigated in this study.

**Study design:**

Research works such as PubMed, Embase, ScienceDirect, Cochrane Library, and two Chinese literature databases (CNKI and WanFang) were searched for studies conducted till August 30th, 2019. Two investigators searched the studies independently, who evaluated the quality of the study using the Newcastle–Ottawa scale (NOS) and extracted data. Pooled standard mean difference (SMD) and diagnostic indexes, with a 95% confidence interval (CI), were calculated using a random-effect model.

**Results:**

Totally, 8 studies involving 473 TAK (208 active and 265 inactive TAK) patients and 252 healthy controls were eventually included in the meta-analysis. PTX3 level in the blood in active TAK patients were found to be higher than that in dormant TAK with pooled SMD of 0.761 (95% CI = 0.38–1.14, *p*<0.0001; I^2^ = 68%, *p* of Q test = 0.003). And there was no publication bias. Among the 8 studies, 5 studies identified active TAK with both PTX3 and CRP. The pooled sensitivity, specificity, and AUC values of PTX3 in active TAK diagnosis were higher than those of CRP (0.78 [95% CI = 0.65–0.87] *vs*. 0.66 [95% CI = 0.53–0.77], *p* = 0.012; 0.85 [95% CI = 0.77–0.90] *vs*. 0.77 [95% CI = 0.56–0.90], *p* = 0.033; 0.88 [95% CI = 0.85–0.90] *vs*. 0.75 [95% CI = 0.71–0.79], *p* < 0.0001). It showed potential publication bias using Egger’s test (*p* of PTX3 = 0.031 and *p* of CRP = 0.047).

**Conclusions:**

PTX3 might be better than CRP in the assessment of TAK activity. Yet, it should be cautious before clinical use for moderate heterogeneity and potential publication bias of the meta-analysis.

## Introduction

Takayasu arteritis (TAK) is a large-vessel vasculitis, predominantly involving the aorta and its major branches, and causes stenosis of the vessel and ischemic syndrome [1]. Delayed diagnosis and deficiency of validated and uniform measures of disease activity, which guide the treatment, are some of the challenges yet to be resolved in TAK clinical practice [2].

C-reactive protein (CRP), an acute-phase reactant (APR), is frequently used besides erythrocyte sedimentation rate (ESR) for monitoring the disease activity of TAK. Nevertheless, several patients whose disease reappears without increased CRP are estimated, which can also intensify in infectious or inflammatory conditions. Therefore, CRP is not sensitive enough and is unambiguous, which calls for designing novel biomarkers that are an immediate requirement.

Pentraxin 3 (PTX3), another APR and CRP, is part of the PTX superfamily. While PTX3 is a long PTX, CRP is a classic short PTX. Unlike CRP that is produced in the liver, PTX3 is generated at local inflammation sites and is primarily produced by macrophages, neutrophils, and dendritic cells as a result of proinflammatory signals. In a meta-analysis, PTX3 levels were found to be high in the case of autoimmune diseases when compared with normal controls [3]. PTX3 levels that were evaluated in vessels were not only reported to be involved in rheumatological diseases such as systemic lupus erythematosus [4] and rheumatoid arthritis [5], but also in vasculitis such as small vessel vasculitis [6] and giant cell arteritis [7]. PTX3 levels in blood were identified in the tissues of patients with an aortic aneurysm, even in the vessel wall of vasa vasorum [8, 9], from which inflammatory lesions originate in TAK [10]. All the above information indicates that PTX3 may be a biomarker for disease activity in TAK patients. However, contradictory results have been shown in the studies by Tombetti et al. and Alibaz-Oner et al. [11, 12].

Since TAK is a rare autoimmune disease, contradictory results on PTX3 true value on disease activity assessment may be due to the small sample size. Therefore, a meta-analysis and literature review was conducted to ascertain the role of PTX3 in TAK activity assessment and then compare the diagnostic values of TAK disease activity between PTX3 and CRP based on the condition that PTX3 is higher in active TAK than in inactive TAK.

## Materials and methods

### Data sources and searches

This meta-analysis was conducted as per the guidelines of the Preferred Reporting Items for Systematic Reviews and Meta-Analyses (PRISMA) Statement. Literature demonstrating serum or plasma levels of PTX3 and CRP in TAK were systemically searched from the databases such as PubMed, Embase, ScienceDirect, Cochrane Library, and two additional Chinese literature databases, namely, CNKI and WanFang, published till August 30th, 2019. A combination of terms and keywords used for data search are as follows: ‘Takayasu arteritis’ or ‘Takayasu's arteritis’ or ‘TAK’ or ‘TA,’ and ‘PTX3’ or ‘pentraxin 3’ or ‘PTX-3’ or ‘pentraxin-3’. Only full-text papers were searched.

### Inclusion and exclusion criteria

Studies with the following criteria were included: (a) TAK was diagnosed definitely; (b) TAK patients were clearly divided into active and inactive groups; (c) Adequate data on serum or plasma levels of PTX3 in active and inactive patients, and healthy controls were available; (d) Availability of detailed data for evaluating sensitivity, specificity, and area under the curve (AUC) of PTX3 and CRP. Studies reported in more than one paper and those with the most subjects were included.

Articles with the following criteria were excluded:(a) duplicate reports; (b) meeting abstracts or review articles or case reports, and (c) studies that were devoid of details on PTX3 or CRP.

### Literature quality and data extraction

Quality assessment was carried out individually by two reviewers using the Newcastle–Ottawa Score (NOS; studies with the value of NOS ≥ 5 were included in the meta-analysis) tool, and data were extracted. Any disagreements were resolved by discussion or with the help of a third investigator.

The data gathered from each eligible paper included the first author’s name, year of publication, country, study type, sample size, source of controls, methods of detection of PTX3, *p* values of the estimated effects, and mean ± standard deviation (SD) or median values and quartiles of PTX3.

### Statistical analysis

Stata 12.0 (StataCorp LP, College Station, TX, USA) was used for performing the meta-analysis. PTX3 level for each study was estimated by the standardized mean difference (SMD) and 95% confidence interval (95% CI), and has been displayed in a forest plot. If data were presented in terms of median and range, the median value was treated as the mean, whereas SD was calculated by using the range to obtain the appropriate values [13]. If data were in quartiles, SD was calculated as 68% of the interquartile range [14].

Sensitivity, specificity, and AUC values of PTX3 and CRP were also calculated and illustrated in summary ROC (SROC) plot. The pooled AUC values were compared between PTX3 and CRP using the Z-test; *p* < 0.05 was considered statistically significant.

Heterogeneity was assessed by way of Cochrane Q test (Chi-square test, χ^2^) and inconsistency index (I^2^). If *p* > 0.05 of the Q test or I^2^ < 50% was considered low heterogeneity, *p* < 0.05 with I^2^ value in the range 50–75% was considered moderate heterogeneity, and *p* < 0.05 with I^2^ values of > 75% were considered as high heterogeneity. If the encompassed studies were of low heterogeneity, the fixed effect model was applied to pool the data; else, the random effect model was applied. To detect the heterogeneity sources, subgroup analysis was carried out as per various criteria of disease activity.

To identify the outlier studies in case of occurrence of high heterogeneity, sensitivity analyses were conducted. Egger’s linear regression and Harbord’s tests were conducted to evaluate potential publication bias.

## Results

### Literature research and characteristics of studies

**Fig 1:**
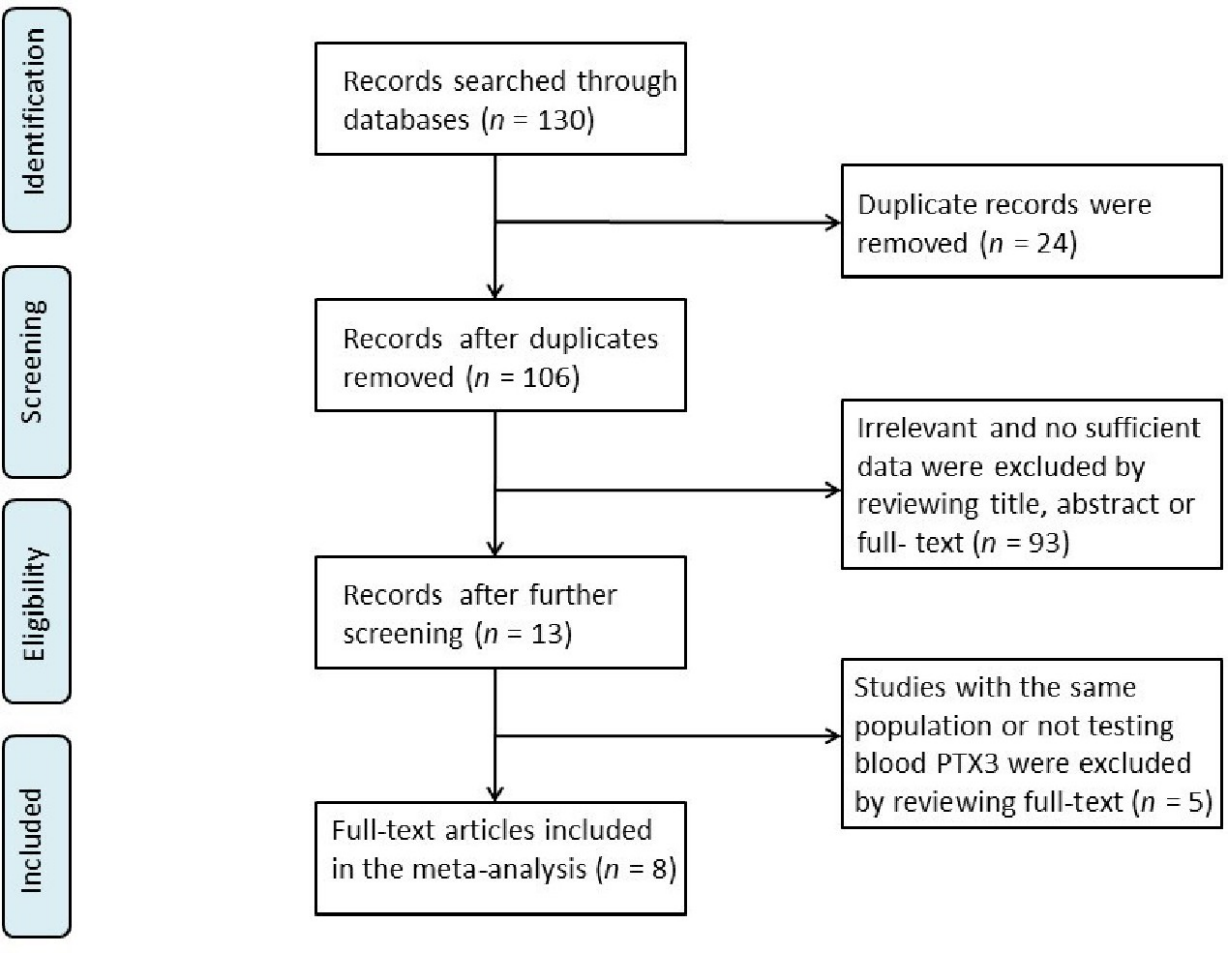
Flow chart of the studies selected in the meta-analysis.

Totally, 130 studies were recognized upon searching using a combination of keywords. Thereafter, eight papers were eventually involved in this meta-analysis following the inclusion and exclusion criteria (Fig 1). These eight studies included 473 TAK patients (208 active and 265 inactive TAK) and 252 healthy controls [8, 9, 11, 12, 15–18]. The aforementioned studies were from Italy and Asia. All the eight studies adopted the criteria provided in 1990 ACR Criteria to diagnose TAK; three criteria were used to measure disease activity. And ELISA test was used in all the studies to measure PTX3 in serum or plasma. The NOS > 5 values were found in all the eight studies, and the related detailed data of each group have been presented in Table 1.

**Table 1:** Characteristics of the eight studies included in the meta-analysis.

References	Country	TAK case		Active TAK		Inactive TAK		Control		*p*	Criteria for active TAK	specimen	NOS
		*n*	Mean ± SD	*n*	Mean ±SD	*n*	Mean ± SD	n	Mean ± SD				
Dagna, 2011	Italy	57	1.35±1.34	27	2.14±1.94	30	0.63±0.12	57	0.11± 0.94	< 0.001	Defined by author	plasma	7
Tombetti, 2014	Italy	42	5.5 (1.3–55)^#^	12	12.0±16.5	30	7.1±3.3	20	NA	0.329	NIH	plasma	7
Ishihara, 2013	Japan	45	6.6±0.61	28	8.3±0.7	17	3.9±0.4	20	3.1± 0.2	< 0.001	NIH	plasma	6
Alibaz-Oner, 2016	Turkey	94	3.5±2.5	25	3.7±2.2	69	3.6±2.5	40	2.5± 1.6	0.981	NIH	plasma	6
Sun, 2013	China	45	18.5±13.9	27	26.7±16.8	18	6.2±4.7	25	0.52± 0.26	< 0.01	NIH	plasma	5
Chen, 2017	China	98	0.32±0.23	45	0.39±0.28	53	0.26±0.16	40	0.18± 0.09	0.004	NIH	serum	6
Wang, 2013	China	60	NA	30	0.088±0.042	30	0.067±0.034	30	0.063±0.038	0.022	NIH	plasma	6
Devarasetti, 2019	India	32	0.51±0.75	14	1.34±1.13	18	0.37±0.25	20	0.32±0.21	< 0.001	PGA	plasma	6

TAK: Takayasu arteritis; NIH: National Institutes of Health; PGA: physician global assessment; NOS: Newcastle–Ottawa Score; NA: not available

^#^ show as median and range.

### Heterogeneity and sensitivity analysis

**Fig 2:**
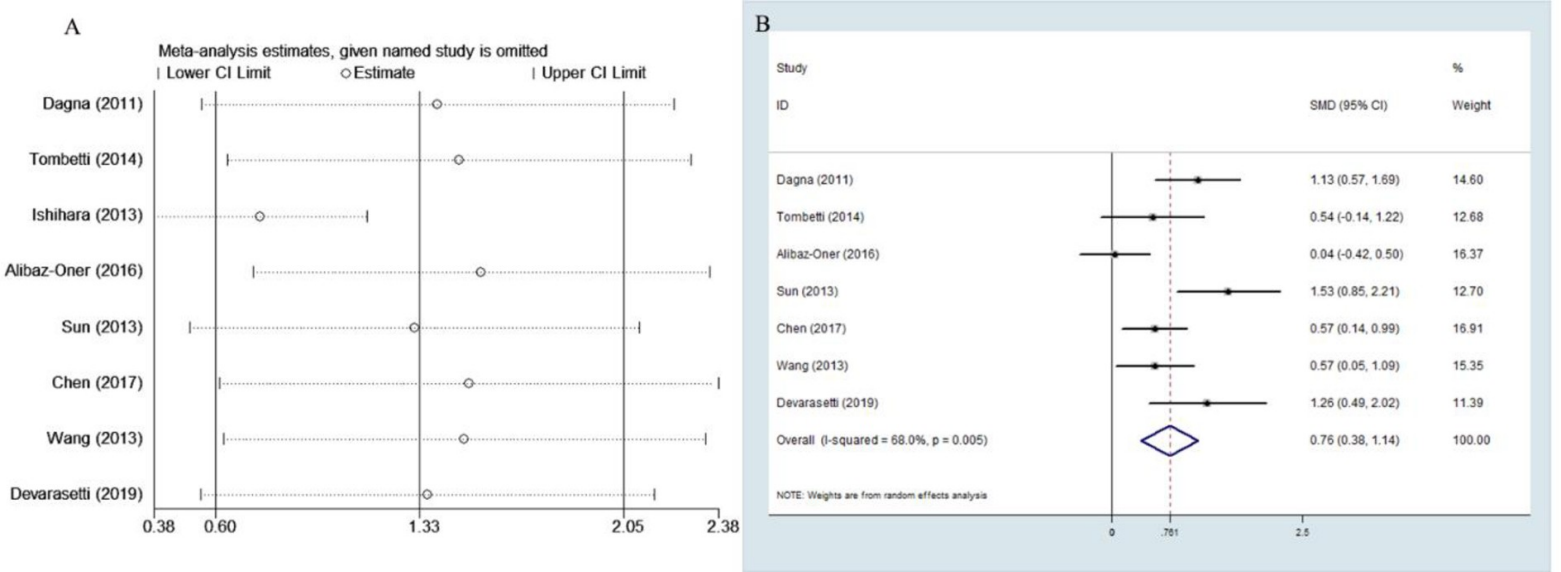
Sensitivity analysis and forest plot. A Sensitivity analysis of the eight studies. B Forest plot of studies on PTX3 level for patients with TAK vs. healthy subjects.

The outcome of the meta-analysis of the eight studies indicated high heterogeneity with *p*<0.05 of the Q test and I^2^ value of 91.2%. Sensitivity analysis showed that the study by Ishihara et al. [9] was the outliner study for its upper 95% CI limit was lower than the estimated pooled SMD. When this study was excluded, I^2^ value was decreased to 68% (Fig 2). Therefore, the rest of the seven studies were included in this meta-analysis.

### Meta-analysis of PTX3 level in active TAK vs. inactive TAK

The random effects model was applied in the meta-analysis for moderate heterogeneity. The outcomes indicated that the PTX3 levels in active TAK were higher than that in quiescent TAK with pooled SMD of 0.761 (95% CI = 0.38–1.14, *p* <0.0001; I^2^ = 68%, *p* of Q test = 0.003; Fig 2). No publication bias was found in performing Egger’s regression test among the seven studies (*p* = 0.09).

### Subgroup analysis

**Fig 3:**
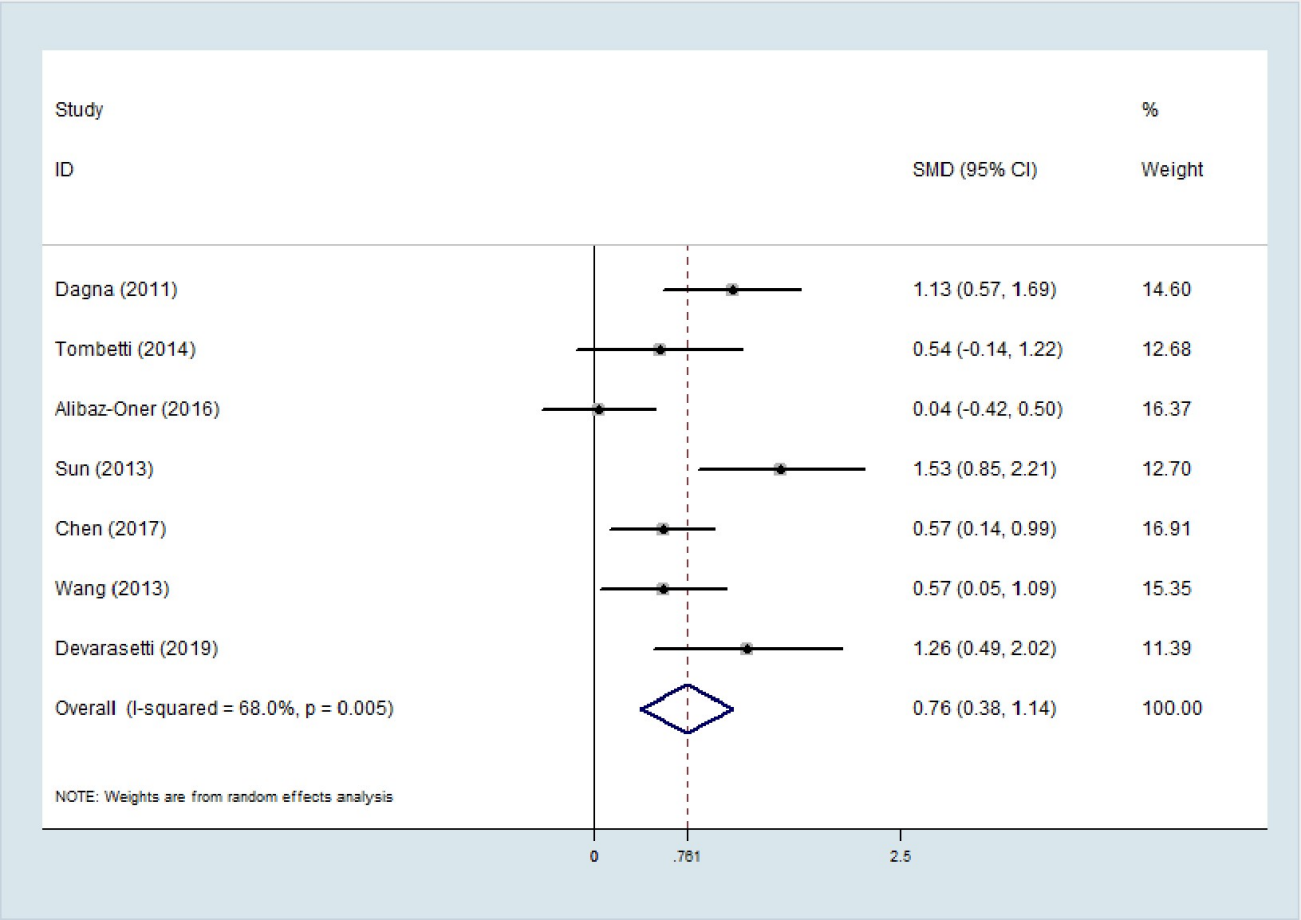
Subgroup analysis by race and active TAK criterion.

Subgroup analysis was conducted by disease criterion to investigate the potential sources of moderate heterogeneity (Fig 3). PTX3 levels in active TAK patients described by the authors, NIH, and PGA criteria were all higher than those in the quiescent TAK patients as per the results. The NIH subgroup's heterogeneity was higher than that of the entire meta-analysis; this result may be attributed to the effect of small sample size or the fact that active TAK defined by NIH includes heterogeneous active disease—both active systemic inflammation and active vascular inflammation. This may be partially demonstrated by the tendency that PTX3 level, reflecting local inflammation, in the active group defined by the author base on strict vascular inflammation was found to be higher than that in the active TAK group by NIH criterion compared with the quiescent group.

### Literature of PTX3 and CRP in diagnostic active TAK

Since PTX3 was evidenced to be higher in active TAK patients than in inactive TAK patients in this meta-analysis, PTX3’s diagnostic values in active TAK assessment was investigated and compared with CRP’s. Five studies that concurrently reported diagnostic indexes of PTX3 and CRP in assessing TAK activity and obtained inconsistent results were included in the study. Their diagnostic parameters cut-off value, AUC value, and *p*-value of comparing their AUC value have been shown in Table 2.

**Table 2:** Characteristics of the five studies included in the meta-analysis of PTX3 and CRP in TAK activity assessment.

References	PTX3								CRP								*p*
	TP	FP	FN	TN	SEN (%)	SPE (%)	AUC	Cut-off (ng/ml)	TP	FP	FN	TN	SEN (%)	SPE (%)	AUC	Cut-off (mg/L)	
Dagna, 2011	24	4	3	26	88.8	86.7	0.919	1	14	8	13	22	51.9	73.3	0.684	6	< 0.05
Ishihara, 2013	23	1	5	16	82.1	94.1	0.949	5.5	20	0	8	17	71.4	100	0.922	2050	NS
Sun, 2013	24	4	3	14	88.9	77.8	0.95	10.71	20	7	7	11	73.1	62.5	0.68	2.9	< 0.05
Chen, 2017	27	11	18	42	57.1	73.1	0.72	0.3071	36	26	9	27	85.0	42.0	0.64	3	0.9252
Devarasetti, 2019	14	1	8	17	64.0	95.0	0.82	0.745	10	2	12	16	46.0	89.0	0.75	17.1	NA

TAK: Takayasu arteritis; PTX3: pentraxin 3; CRP: C-reactive protein; AUC: area under the curve; TP: true positive; FP: false positive; FN: false negative; TN: true negative; SEN: sensitivity; SPE: specificity; NS: Not significant; NA: not available.

### Comparison between PTX3 and CRP in the accuracy of TAK activity assessment

**Fig 4:**
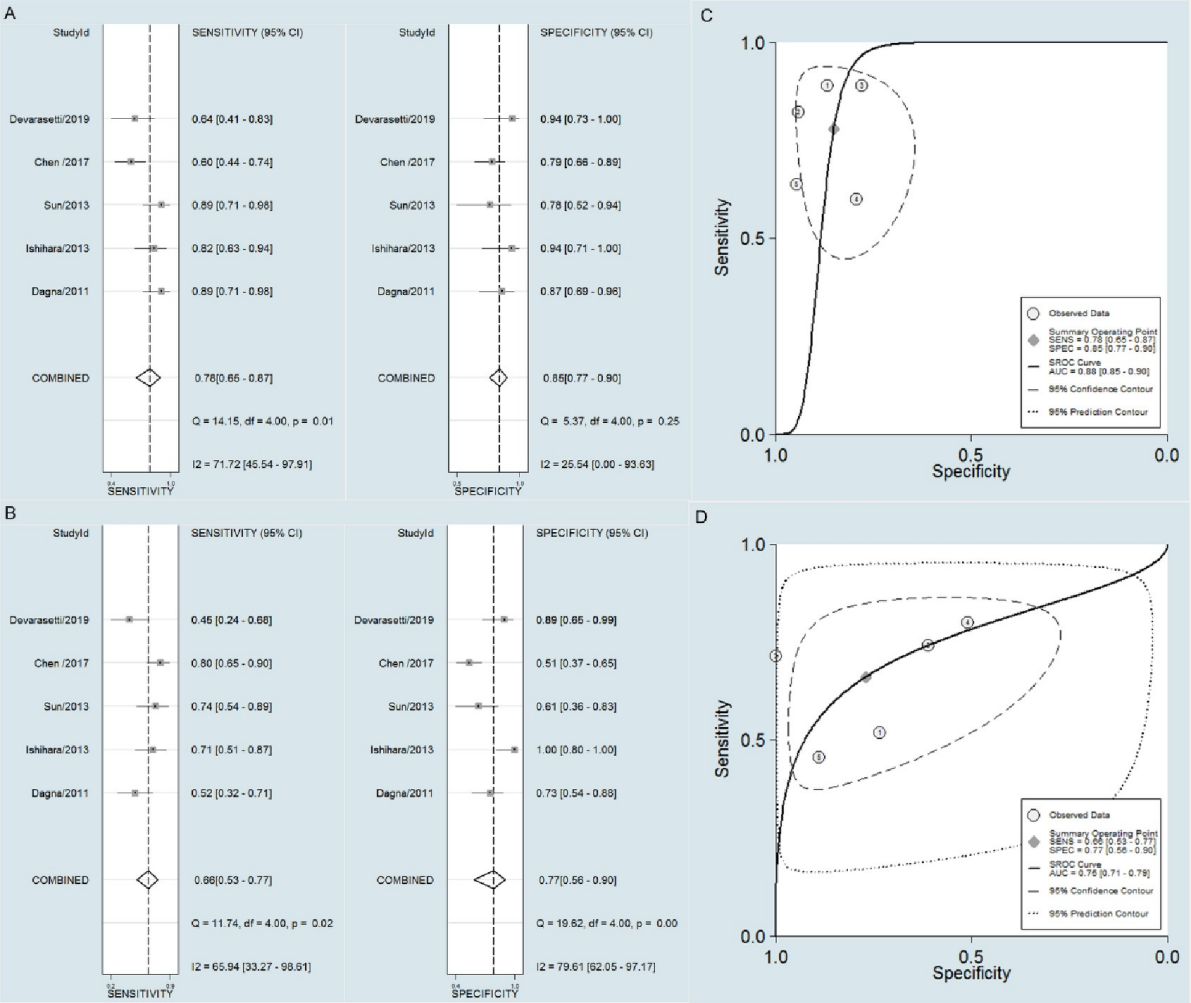
Forest plot of the sensitivity and specificity, and SROC. Forest plot of the sensitivity and specificity of PTX3 (A) and CRP (B). SROC of the accuracy of PTX3 (C) and CRP(D) in TAK activity assessment.

A bivariate random effects model was utilized in this meta-analysis. The pooled sensitivity, specificity, and AUC values of PTX3 in active TAK diagnosis were observed to be higher than those of CRP (0.78 [95% CI = 0.65–0.87] *vs*. 0.66 [95% CI = 0.53–0.77], *p* = 0.012;0.85 [95% CI = 0.77–0.90)] *vs*. 0.77 [95% CI = 0.56–0.90], *p* = 0.033; 0.88 [95% CI = 0.85–0.90] *vs*. 0.75 [95% CI = 0.71–0.79], *p*< 0.0001; Fig 4)).

The outcomes of this meta-analysis were stable, and no studies were excluded. No small-study effects or publication bias were found among the five studies by Harbord’s test (*p* of PTX3 = 0.124 and *p* of CRP = 0.478). However, when we adopted a more sensitive test, Egger’s test, because of not low heterogeneity, it showed publication bias (*p* of PTX3 = 0.031 and *p* of CRP = 0.047)

## Discussion

Strong evidence suggests that serum or plasma PTX3 levels are greater in TAK patients. This meta-analysis indicated that the PTX3 level in active TAK was remarkably higher than that in inactive TAK with moderate heterogeneity among the seven explored studies. The ethnic difference may contribute to heterogeneity; however, there were not enough studies of each population to do subgroup analysis. Ramirez et al. [19] confirmed that the activity of vasculitis was the principal variable affecting the PTX3 levels. Therefore, the disease activity criterion may be speculated to be the potential influencing factors for heterogeneity. We performed subgroup analysis and found that various activity criteria may be attributable partly to the heterogeneity. Five studies included in the meta-analysis had adopted the NIH criterion or the so-called Kerr’s criterion [20], which is significantly used in measuring the TAK activity. PTX3 levels reflecting local inflammation may be influenced by NIH criteria, which does not identify vascular inflammation or systemic inflammation. This study reveals that when strict criterion defined as new-onset or worsened vascular lesions was used [8], PTX3 levels in the active TAK by the criterion seemed to be greater than those evaluated by NIH criterion. However, it should be cautious of the small sample size. This was also demonstrated in a study by Tombetti et al. Similar levels of PTX3 in active TAK and quiescent TAK patients were found. A higher level was still recorded in patients with vascular inflammation detectable through imaging than in patients with no evident vessel inflammation [11].

Besides being involved in inflammation, PTX3 has also been evidenced to play critical regulatory roles in endothelial and smooth muscle cell function, which dominates the mechanism of vascular remodeling in TAK patients. Angiogenesis occurs in the arterial wall in active TAK [21] and has been evidenced by proliferated endothelial cells [22], which aids the development of collateral circulation of the arterial wall. PTX3 inhibits angiogenesis [23] by binding and neutralizing the fibroblast growth factor 2 (FGF2). It can decrease endothelial permeability [24] while binding vascular endothelial growth factor (VEGF). Nevertheless, opposite results also exist that indicate that PTX3 promoted angiogenesis [25]. On knocking out of PTX3, VEGF receptor 2 (VEGFR2) expression and endothelial cell proliferation were recorded to be decreased [26]. PTX3 might modulate angiogenesis through a complex molecular regulation network, as evidenced by a certain study. Moreover, PTX3 is the most potent predictor of inflammation-induced neointimal thickening after vascular injury [27]. Contrarily, PTX3 is assumed to inhibit intimal hyperplasia, as its deficiency tends to aggravate intimal hyperplasia by promoting proliferation and migration of smooth muscle cells after vascular injury [28]. Nonetheless, through a 5-year follow-up, it could be established that plasma levels of PTX3 predict neither intima-media thickness progression nor incidence of cardiovascular events in the general population [29]. Collectively, PTX3 just acts as an indicator of the inflammatory response and may be linked to the vessel wall's angiogenesis but fails to explain intima thickening and vessel stenosis in TAK.

PTX3 was evidenced in this meta-analysis to be more accurate than CRP for differentiating between active and inactive TAK according to NIH criterion or the author among five studies that detected the two indicators simultaneously. Following may be the reasons. First, since PTX3 is manufactured at local inflammation sites and CRP mainly in the liver, PTX3 tends to reflect local inflammation, including local arteries. In contrast, CRP indicates the burden of systemic inflammation [8]. This may also be why PTX3 levels are not linked to CRP in TAK [8, 11, 15, 17]. Second, PTX3 level increases prior to that of CRP in response to infective or inflammatory stimuli: PTX3’s peak time is 6–8 h, whereas CRP’s being 24–30 h [30]. Eventually, PTX3 changes better reflected endothelial function than CRP assessed by flow-mediated dilation [31].

While steroids are usually very effective in clinical treatment and are known to decrease CRP, PTX3’s response to steroids is unclear. PTX3 levels were not found to be correlated to prednisolone dose in certain research [9, 32]. In contrast, a study by Ramirez et al. confirmed PTX3 levels to be correlated with the prednisolone dose. However, the PTX3 levels were mainly affected by active vasculitis, but not the prednisolone dose [20]. In addition, methotrexate seems not to affect PTX3 level in TAK, and it was proved not to influence PTX3 in inflammatory rheumatic disease [12, 33]. However anti-IL-6 showed contrary results. While TNF-α or IL-6-receptor inhibitors therapy was reported to reduce the production of PTX3, reflecting vascular inflammation and progression in TAK patients [11], IL-6-receptor antibody improved neither vascular stenosis nor thickness nor PTX-3 level [32]. Thus it is suspected that the ability to reduce PTX3 of IL-6-receptor inhibitors might depend on whether it can alleviate vascular injury.

Despite the sample size being large for the rare autoimmune disease(s) in this meta-analysis, the only limited population was included in the study. And it has some other limitations. First, all the included studies were carried out on TAK patients with known disease activity; thus, the PTX3 value on predicting disease activity derived in this meta-analysis should be used cautiously. Second, factors influencing high heterogeneity were unclear. Treatment may account partially for high heterogeneity; however, adequate data on the prednisolone dose or biologicals in each included study was lacking. Third, there were potential small-study effects so that more publications, especially those with negative results, should be concentrated on in the future.

## Conclusion

To conclude, this meta-analysis found circulating PTX3 is significantly increased in active TAK compared with inactive TAK. And PTX3 reflects TAK activity better than CRP. Nevertheless, the present study has high heterogeneity and potential publication bias. It should be cautious when PTX3 is generally used as a biomarker for assessing TAK in clinics. And factors such as glucocorticoid therapy and various activity criteria possibly influencing PTX3 levels, should be further investigated before clinical use.

## Supporting information

S1 Table PRISM (Preferred Reporting Items for Systematic Reviews and Meta-Analyses) 2009 checklist. (DOCX)

S2 Table The full search strategies. (DOCX)

S3 Table Data of eight studies included in the meta-analysis. (XLSX)
