# Supplementary material for: Pentraxin 3 is more accurate than C-reactive protein for Takayasu arteritis activity assessment: A systematic review and meta-analysis
Source: PLoS One. 2021 Feb 2;16(2):e0245612. doi: 10.1371/journal.pone.0245612 (PMC7853471; doi:10.1371/journal.pone.0245612)
Supplement: S2 Table — (DOCX) [file pone.0245612.s002.docx]

**S2 Table Full search strategies.**

| **Database** | **Syntaxis** |
| --- | --- |
| PubMed | (“Takayasu arteritis” OR “Takayasu's arteritis” OR TAK OR TA) AND (“pentraxin 3” OR “pentraxin-3” OR PTX3 OR PTX-3) |
| Embase | (“Takayasu arteritis” OR “Takayasu's arteritis”) and (“pentraxin 3” OR “pentraxin-3”) |
| ScienceDirect | (“Takayasu arteritis” OR “Takayasu's arteritis”) and (“pentraxin 3” OR “pentraxin-3”) |
| Cochrane Library | All Test (“Takayasu arteritis” OR “Takayasu's arteritis” OR TAK OR TA) AND (“pentraxin 3” OR “pentraxin-3” OR PTX3 OR PTX-3) |
